# LILRB4 regulates the function of decidual MDSCs via the SHP-2/STAT6 pathway during *Toxoplasma gondii* infection

**DOI:** 10.1186/s13071-023-05856-4

**Published:** 2023-07-17

**Authors:** Yuantao Li, Jingjing Guo, Haixia Zhang, Zhidan Li, Yushan Ren, Yuzhu Jiang, Xianbing Liu, Xuemei Hu

**Affiliations:** 1 Department of Immunology, Binzhou Medical University, Yantai, 264003 Shandong People’s Republic of China; 2 Department of Gynecology and Obstetrics, Yantai Affiliated Hospital of Binzhou Medical University, Yantai, Shandong People’s Republic of China

**Keywords:** *Toxoplasma gondii*, Fetal, Maternal tolerance, Decidual MDSCs, LILRB4, Dysfunction

## Abstract

**Background:**

*Toxoplasma gondii* infection can cause adverse pregnancy outcomes, such as recurrent abortion, fetal growth restriction and infants with malformations, among others. Decidual myeloid-derived suppressor cells (dMDSCs) are a novel immunosuppressive cell type at the fetal-maternal interface which play an important role in sustaining normal pregnancy that is related to their high expression of the inhibitory molecule leukocyte immunoglobulin-like receptor B4 (LILRB4). It has been reported that the expression of LILRB4 is downregulated on decidual macrophages after *T. gondii* infection, but it remains unknown whether *T. gondii* infection can induce dMDSC dysfunction resulting from the change in LILRB4 expression.

**Methods:**

LILRB4-deficient (LILRB4^−/−^) pregnant mice infected with *T. gondii* with associated adverse pregnancy outcomes, and anti-LILRB4 neutralized antibodies-treated infected human dMDSCs were used in vivo and in vitro experiments, respectively. The aim was to investigate the effect of LILRB4 expression on dMDSC dysfunction induced by *T. gondii* infection.

**Results:**

*Toxoplasma gondii* infection was observed to reduce STAT3 phosphorylation, resulting in decreased LILRB4 expression on dMDSCs. The levels of the main functional molecules (arginase-1 [Arg-1], interleukin-10 [IL-10]) and main signaling molecules (phosphorylated Src-homology 2 domain-containing protein tyrosine phosphatase [p-SHP2], phosphorylated signal transducer and activator of transcription 6 [p-STAT6]) in dMDSCs were all significantly reduced in human and mouse dMDSCs due to the decrease of LILRB4 expression induced by *T. gondii* infection. SHP-2 was found to directly bind to STAT6 and STAT6 to bind to the promoter of the Arg-1 and IL-10 genes during *T. gondii* infection.

**Conclusions:**

The downregulation of LILRB4 expression on dMDSCs induced by *T. gondii* infection could regulate the expression of Arg-1 and IL-10 via the SHP-2/STAT6 pathway, resulting in the dysfunction of dMDSCs, which might contribute to adverse outcomes during pregnancy by *T. gondii* infection.

**Graphical abstract:**

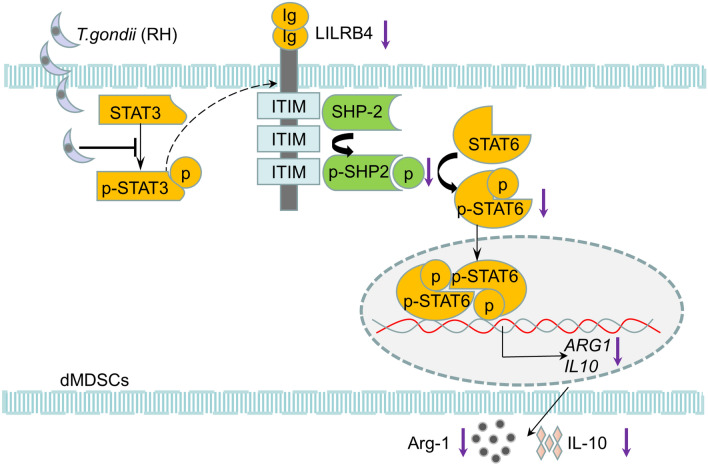

**Supplementary Information:**

The online version contains supplementary material available at 10.1186/s13071-023-05856-4.

## Background

*Toxoplasma gondii* is an obligate intracellular protozoan parasite that can infect almost all warm-blooded animals. It is estimated that about 30% of the human population is affected by this parasite worldwide [1]. Toxoplasmosis is a life-threatening infection in immunosuppressed individuals; in this context, infected pregnant women often experience recurrent abortion, preterm birth, stillbirth, fetal growth restriction and infants with malformations [2–5]. Several studies have shown that *T. gondii* infection during pregnancy leads to the dysfunction of decidual immune cells at the maternal–fetal interface, which causes the disruption of the immune microenvironment [6–9]. The immune microenvironment comprises various decidual immune cells, such as decidual natural killer (dNK) cells, decidual macrophages (dMφ), decidual dendritic cells (dDCs) and decidual myeloid-derived suppressor cells (dMDSCs), all of which play an important role in sustaining normal pregnancy [10]. MDSCs are a heterogeneous population of cells consisting of myeloid progenitor cells and immature myeloid cells [11, 12]. In humans, MDSCs are defined as cells of the CD33^+^ human leukocyte antigen DR (HLA-DR)^neg/l^°^w^ phenotype [13]. In mice, MDSCs are characterized by the co-expression of CD11b^+^ Gr-1^+^ [14]. Healthy pregnant women have more MDSCs in their peripheral blood than nonpregnant women, and women who have had a miscarriage have fewer MDSCs in their blood and endometrium than healthy pregnant women [15]. In addition, the accumulation of MDSCs on the maternal–fetal interface during gestation has been directly implicated in the maintenance of maternal–fetal tolerance [16, 17]. When compared to healthy pregnant mice, mice that respond to abnormal delivery have fewer MDSCs in their placenta and uteri [18, 19]. All of these findings suggest that MDSCs are closely related to a successful pregnancy and are essential for maternal–fetal immune tolerance. More recently, MDSCs were observed in human gestation, and they seemed to play an important role in suppressing CD4^+^ and CD8^+^ T-cell proliferation [20]. Studies have shown that the immunosuppressive capacity of MDSCs is adjusted by the inhibitory molecule leukocyte immunoglobulin-like receptor B4 (LILRB4) in patients with non-small-cell lung cancer and in mice with melanoma occurring as subcutaneous tumors [21, 22]. LILRB4 belongs to the leukocyte immunoglobulin G (Ig)-like receptor superfamily and is expressed in a wide variety of immune cell types, including DCs, Mφ and MDSCs [22, 23]. This molecule consists of an extracellular domain with two Ig-like domains, a transmembrane segment and a cytoplasmic domain with three immunoreceptor tyrosine-based inhibitory motifs (ITIMs) [24, 25]. LILRB4 expressed on MDSCs has been shown to regulate the immunological function of MDSCs and facilitate tumor immune tolerance [26]. Our previous studies have shown that the downregulation of LILRB4 expressed on dMφ and dDCs may contribute to the adverse outcomes during pregnancy caused by *T. gondii* infection [9, 27]. In addition, the results of one study implied that the expression of LILRB4 was mediated by the phosphorylation of signal transducer and activator of transcription 3 (STAT3) in human vascular endothelial cells [28]. However, further research is required to determine whether *T. gondii* infection can affect LILRB4 expression in dMDSCs and characterize the molecular mechanism of regulation.

Some studies have reported that two intracellular ITIMs of LILRB4 interact with Src-homology 2 domain-containing protein tyrosine phosphatase (SHP-2) and activate the phosphorylation of SHP-2 [29, 30], and that the SHP-2 domain recruits STAT6 and stimulates the phosphorylation of signal transducer and activator of transcription 6 (STAT6) followed by its dimerization [31]. The phosphorylated STAT6 (p-STAT6) accumulates in the nucleus of macrophages and regulates the expression of arginase-1 (Arg-1) [32]. According to several studies, STAT6 activation (p-STAT6) in M2 macrophages of patients with acute liver failure and in the cerebrospinal fluid of those with primary central nervous system lymphoma significantly correlates with the presence of interleukin -10 (IL-10) [33, 34]. Results from our previous study showed that the downregulation of LILRB4 on dMφ induced by *T. gondii* infection could result in the decrease of Arg-1 and IL-10 [27]. However, more research is necessary to determine whether the change in LILRB4 expression level during *T. gondii* infection influences the expression of Arg-1 and IL-10 on dMDSCs via the SHP-2/STAT6 pathway.

In the present study, human dMDSCs and LILRB4^−/−^ pregnant mice were utilized to investigate the change in LILRB4 expression on dMDSCs during *T. gondii* infection and explain the molecular mechanism of the change in LILRB4 expression, leading to the immunosuppressive dysfunction of dMDSCs.

## Methods

### *Toxoplasma gondii*, RH strain

The tachyzoites of the *T. gondii* RH strain were harvested from human foreskin fibroblast (HFF) cells grown in 10% fetal bovine serum (FBS) and 100 IU/ml penicillin/streptomycin. After co-culture of HFF cells and toxoplasma tachyzoites for 48 h, the cultures were collected, the cells removed by centrifugation at 433 *g* for 5 min and the tachyzoites remaining in the supernatant purified by centrifugation at 2810 *g* for 7 min.

### Experimental animals and* T. gondii* infection

Wild-type (WT) C57BL/6 mice (8- to 10 week-old females and males) were purchased from Pengyue Laboratory Animal Technology Co., Ltd. (Jinan, China). LILRB4-deficient (LILRB4^−/−^) C57BL/6 mice were obtained from Bioray Laboratories Inc. (Shanghai, China). All mice were maintained in a specific pathogen-free facility at 23 °C ± 2 °C, 55% ± 5% relative humidity and a 12/12-h (light/dark) cycle, with access to abundant sterilized water and food (Jiangsu Biological Engineering Co., Ltd., China). The females with a vaginal plug were designated as gestational day (Gd) 0 of pregnancy following overnight cohabitation with males. WT pregnant C57/BL6 mice were randomly divided into three groups: (i) an uninfected group; (ii) an infected group; and (iii) an infected group treated with the STAT3 inhibitor cucurbitacin I (synonym: JSI-124; MCE Technologies, Suzhou, China). LILRB4^−/−^ pregnant mice were utilized as the infected LILRB4^−/−^ group. Each group included 10 mice.

To explore the molecular mechanism of LILRB4 regulating the function of decidual MDSCs in adverse pregnancy outcomes caused by *T. gondii* infection early in the first trimester (from 0 to 7 day [35]), pregnant mice in the infected group received an intraperitoneal (i.p.) injection of 200 tachyzoites in 200 μl sterile phosphate-buffered saline (PBS) on Gd 7. The uninfected group received an i.p. injection of 200 μl sterile PBS only. Fetuses, uteri and placentas were harvested on Gd 13 for analysis, and the number of dMDSCs [36] and the expression level of LILRB4 [9, 37] were determined during pregnancy.

For JSI-124 treatment, WT pregnant mice were infected with *T. gondii* as described above and then i.p. injected with 1 mg/kg body weight of JSI-124 3 times a week (Gd 7, Gd 9 and Gd 11). The infected WT pregnant mice received an i.p. injection with 200 μl sterile PBS only.

The experimental protocols used in all experiments adhered to all relevant ethical regulations for animal testing and research and were approved by the Institutional Animal Care and Research Advisory Committee of Binzhou Medical University, China (permit number: 2017-009-09). The use of animals in our study was in accordance with the guidelines issued by the Chinese Council on Animal Care.

### Genotyping of LILRB4^−/−^ mice

Genomic DNA was extracted from mouse tails, and complementary DNA (cDNA) was synthesized by PCR, as described previously [27]. After initial denaturation (30 s at 95 °C), PCR was performed with 40 amplifcation cycles of denaturation for 10 s at 95 °C, annealing for 30 s at 65 °C, and extension for 5 s at 72 °C, followed by a final extension for 3 min at 72 °C. The PCR products were run on gels and stained with GelStain (Transgene S.A., Illkirch-Graffenstaden, France) to visualize DNA. Primers for PCR amplification were LILRB4 P1 (5′-ACCGGTGGATGTGGAATGTGTG-3′), LILRB4 P2 (5′-GTCCTGGGTTCCAGAATAAGAC-3′) and LILRB4 P3 (5′-TCTGCTCTTAGGAAATTACAGAA-3′). The expected PCR product sizes were 260 bp (mutant), 371 and 260 bp (heterozygote) and 371 bp (wild-type).

### Cell preparation of mice

The experimental protocol was performed as previously described [17] with modifications. The uteri and placentas of the mice were carefully separated and washed several times with cold PBS, following which the tissues were cut into small pieces and digested with 0.1% collagenase type IV (Sigma-Aldrich, St. Louis, MO, USA) and 25 IU/ml DNase I (Sigma-Aldrich) for 1 h at 37 °C with shaking. The digested pieces were filtered through 48-μm sterile mesh. Mononuclear cells were collected from the white film layer after Ficoll density gradient centrifugation in mouse lymphocyte separation medium (TBD Science, Tianjin, China). As a last step, the cells were collected, resuspended in cold PBS and utilized for subsequent flow cytometry analysis.

### Collection of human samples

Clinical samples of decidual tissues were provided by the Department of Obstetrics and Gynecology of Yantai Affiliated Hospital associated with Binzhou Medical University and by the Yantai Zhifu District Maternal and Child Health Hospital. Aborted tissues from pregnant women who had undergone healthy voluntary abortions during the first trimester of pregnancy (gestational age: 6–10 weeks) were aseptically collected and washed 5 to 8 times with PBS under sterile conditions. Decidual tissues were delivered to our laboratory within 2 h of the abortion and kept in Dulbecco’s Modified Eagle Medium (DMEM)/high-glucose medium (HyClone, GLogan, UT, USA) with 100 IU/ml penicillin and 100 μg/ml streptomycin (Sigma-Aldrich).

Prior to beginning the study, written informed consent was obtained from all the participants or their legal guardians. The sample collection procedure was approved by the Ethics Committee of Binzhou Medical University (Approval Number: 2017-016-01).

### Isolation and purification of human dMDSCs

The decidual tissues were washed with cold PBS, cut into small fragments using ophthalmic scissors and digested with 0.1% collagenase IV (Sigma-Aldrich) and 25 IU/ml DNase I (Sigma-Aldrich) in an incubator at 37 °C for 60 min. The resulting suspension was filtered through 48-µm mesh and washed twice in cold PBS. The decidual mononuclear cells used for flow cytometry were collected from the white film layer after Ficoll density gradient centrifugation using human lymphocyte separation medium (TBD Science). To remove decidual macrophages and stromal cells, mononuclear cells were cultured at 37 °C for 1 h, and cells were collected in the supernatant. These cells were used for flow cytometry staining and analysis. The dMDSCs were purified using a human CD33-positive isolation kit and HLA-DR-negative selection kit (Stemcell, Canada) according to the manufacturer’s instructions. The purified dMDSCs were analyzed using Western blot, quantitative reverse transcription PCR (qRT–PCR), co-immunoprecipitation (Co-IP) and chromatin-immunoprecipitation (ChIP) assays. The specific culture and treatment conditions of the purified dMDSCs are explained for each experiment.

### Human decidual mononuclear cell culture and treatment

The mononuclear cells were divided equally into two groups: uninfected and infected groups. *Toxoplasma gondii* tachyzoites were added to the cells of the infected group at a ratio of 1:5 (*T. gondii* tachyzoites:cells). All cells were cultured in RPMI 1640 medium supplemented with 10% FBS (Gibco, Thermo Fisher Scientific, Waltham, MA, USA), 100 IU/ml streptomycin and 100 IU/ml penicillin for 24 h at 37 °C in a humidified 5% CO_2_ incubator. All of the cells were prepared for flow cytometry staining and analysis.

### Flow cytometry staining and analysis

The prepared human and murine decidual mononuclear cell (1 × 10^6^ cells of each group) suspensions were first stained with membrane molecules and then with intracellular molecules, such as Arg-1, IL-10, phosphorylated SHP2 (p-SHP2) or p-STAT6, after the cells were treated with a membrane rupture kit according to the manufacturer’s instructions (eBioscience, San Diego, CA, USA). The experiments were replicated at least 8 times. Cells were analyzed using a BD FACSCanto™ TM II Flow Cytometer (BD Biosciences, Franklin Lakes, NJ, USA) and using FlowJo analysis software (FlowJo LLC, Ashland, OR, USA). The gating strategies are provided in Additional file 1: Figure S1.

### Western blot analysis

Purified human dMDSCs were infected with *T. gondii* tachyzoites at a 1:5 ratio (*T. gondii*tachyzoites: cells) with or without 10 μM Static (STAT3 inhibitor in vitro [STAT3i]), 10 μg/ml anti-LILRB4 (αLILRB4) neutralizing antibody (Invitrogen, Thermo Fisher Scientific), 500 ng/ml recombinant human apolipoprotein E (APOE), 10 μM SHP099 (SHP-2 inhibitor [SHP-2i]) or 100 nM AS1517499 (STAT6 inhibitor [STAT6i]). About 2 × 10^7^ dMDSCs of each group were cultured in RPMI 1640 medium supplemented with 10% FBS (Gibco, Thermo Fisher Scientific) and 100 IU/ml penicillin/streptomycin (Sigma-Aldrich) for 24 h at 37 °C in a humidified 5% CO_2_ incubator. All of the cultured human dMDSCs were harvested, lysed with RIPA lysis buffer (Beyotime Biotechnology, Nantong, China) and then centrifuged at 13,400 *g* for 20 min at 4 °C. After the concentration was measured, equal amounts of protein were loaded into 10% or 12% sodium dodecyl sulfate-polyacrylamide gel electrophoresis (SDS–PAGE) gels and the products were then transferred to polyvinylidene fluoride (PVDF) membranes (MilliporeSigma, Burlington, MA, USA). The membranes were blocked at room temperature for 2 h in 5% skim milk in TBS-T buffer and then incubated overnight at 4 °C with primary antibodies on a shaker. Next, the membranes were incubated with horseradish peroxidase (HRP)-labeled secondary antibodies (Abmart Inc., Berkeley Heights, NJ, USA) at 37 °C for 1 h. The hybridization signal bands were visualized using an enhanced chemiluminescence (ECL) detection kit (Yeasen Biotechnology, Shanghai, China). Protein expression levels were determined using Image J software. The experiments were replicated 5 times.

### Co-immunoprecipitation

To investigate the interaction between SHP-2 and STAT6, purified human dMDSCs were infected with *T. gondii* tachyzoites at a ratio of 1:5 (*T. gondii* tachyzoites:cells) for 24 h with or without 500 ng/ml APOE. About 2 × 10^7^ cells of each group were collected and washed twice with PBS. All cells were lysed with IP Binding Buffer (Solarbio Life Science, Beijing, China) supplemented with phenylmethylsulfonyl fluoride (PMSF). After 20 min on ice, the supernatant was centrifuged at 14,000 *g* at 4 °C, following which the protein was extracted and quantified: 15% of the whole-cell lysis was prepared for input with 5× loading buffer boiling for 10 min at 100 °C. After being preabsorbed with protein, A/G agarose beads (Solarbio Life Science) together with anti-STAT6 antibody (Abcam, Oxford, UK) for 2 h at 4 °C with shaking, the remaining extracts were incubated with antibody-conjugated protein A/G magnetic beads overnight with shaking at 4 °C. Isotype IgG was used as a negative control. Beads were washed 4 times with immunoprecipitation washing buffer (Solarbio Life Science) and denatured at 100 °C for 10 min. Protein samples were used for Western blot analyses. Equal amounts of extracts were separated by SDS–PAGE, and the products were transferred onto nitrocellulose membranes and blotted with specific antibodies.

### Reagents and antibodies

The reagents and antibodies used in the study are listed in Additional file 2: Table S1.

### Quantitative RT‒PCR

Total RNA from human dMDSCs was extracted with TRIzol reagent (Invitrogen, Thermo Fisher Scientific), and cDNA was synthesized using a SuperRT cDNA Synthesis Kit (CoWin BioSciences, Cambridge, MA, USA) according to the manufacturer’s recommended protocol. All messenger RNA (mRNA) expression levels were analyzed by qRT‒PCR using an UltraSYBR One Step RT‒qPCR Kit (CoWin BioSciences) in a Bio-Rad iQ5 multicolor RT‒PCR system (Bio-Rad Laboratories, Hercules, CA, USA). Glyceraldehyde 3-phosphate dehydrogenase (GAPDH) was used for the normalization of mRNA expression. The experiments were replicated 6 times. The primers used for the RT-qPCR are shown in Additional file 2: Table S1. The relative change in the expression of *LILRB4* was estimated using the 2^−ΔΔCT^ method.

### ChIP assay

The STAT6 binding regions on the promoters of the genes for Arg-1 and IL-10 (*ARG1* and *IL10*) were predicted with the NCBI JASPAR database online. The primers used for ChIP-qPCR were synthesized by Sangon Biotech and are listed in Additional file 2: Table S1.

The ChIP experiment was carried out using a SimpleChIP® Enzymatic Chromatin IP Kit (Cell Signaling, Danvers, MA, USA) according to the manufacturer’s instructions. For cross-linking of proteins to DNA, about 2 × 10^7^ cells from the uninfected group and the infected group were treated with 1% paraformaldehyde at room temperature for 10 min; then glycine was added to a final concentration of 0.125 M, and the sample was incubated for 5 min at room temperature to stop cross-linking. After washing twice with ice-cold PBS, the cells were digested to an optimal DNA length of approximately 150–900 bp by using a lysis buffer and sonication (sonication at 20% power: 5 s ON/10 s OFF for 5 min on ice). For immunoprecipitation, protein–DNA complexes were incubated overnight with an antibody with shaking at 4 °C, following which protein G agarose beads were added, and the samples were incubated for 2 h at 4 °C with shaking. Protein G agarose beads were washed 4 times using a low-salt washing buffer and 2 times using a high-salt washing buffer at 4 °C for 5 min with shaking. DNA was eluted in ChIP elution buffer, reversed cross-linked by incubation in 5 M NaCl and proteinase K for 2 h at 65 °C, purified via DNA wash buffer and purification collection tube and subjected to qPCR. The DNA was analyzed by qPCR using an UltraSYBR One-Step RT-qPCR Kit (CoWin BioSciences) in a Bio-Rad iQ5 multicolor RT‒PCR system (Bio-Rad Laboratories). The experiments were replicated 3 times.

### Statistical analysis

Statistical analyses were performed using GraphPad Prism 8 software (GraphPad Software, La Jolla, CA, USA). Data are presented as the mean ± standard error (SE). Unpaired and paired *t*-tests were used to identify the differences. One-way analysis of variance was applied with 95% confidence intervals. Significance was defined as *p* < 0.05.

## Results

### LILRB4 is decreased in dMDSCs after *T. gondii* infection

**Fig. 1:**
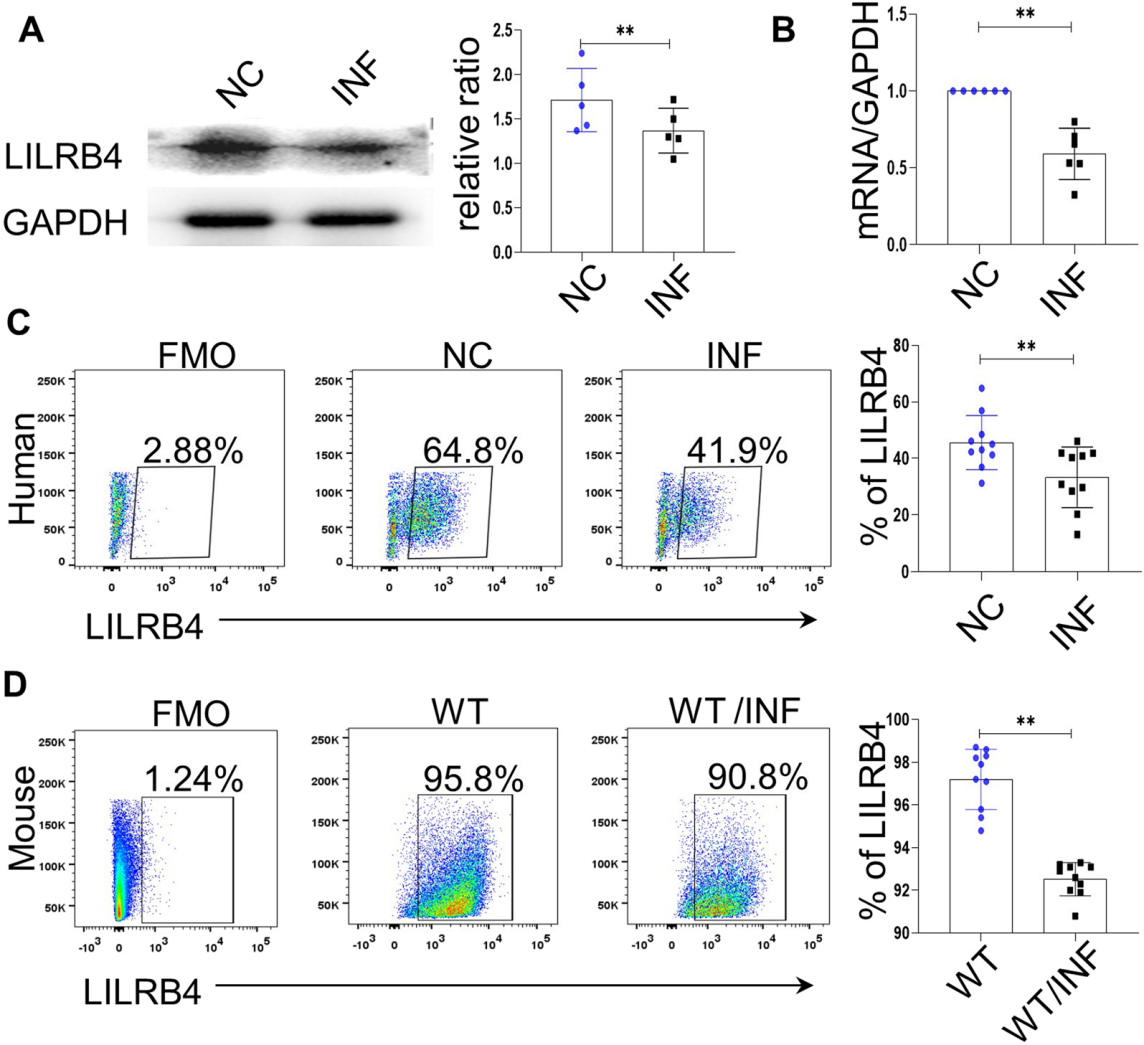
The expression level of LILRB4 in dMDSCs was decreased after *Toxoplasma gondii* infection. The expression level of LILRB4 on human decidual myeloid-derived suppressor cells (dMDSCs) was analyzed in the uninfected and infected groups by Western blot (**a**), qRT–PCR (**b**) and flow cytometry (**c**). **d** The levels of LILRB4 on mouse dMDSCs from the WT and infected mice were analyzed by flow cytometry. The human data were identified by a paired *t*-test; the mouse data were identified by the unpaired *t*-test. Asterisks indicate significant difference at *p < 0.05, ** *p* < 0.01. FMO, fluorescence minus one; GAPDH, glyceraldehyde 3-phosphate dehydrogenase; INF, infected; LILRBA, leukocyte immunoglobulin-like receptor B4; mRNA, messenger RNA; NC, negative control; WT, wild type.

To explore the change in the expression of LILRB4 on dMDSCs during *T. gondii* infection, we detected LILRB4 expression levels on human dMDSC using Western blot, qPCR and flow cytometry analyses. We also detected LILRB4 expression levels on mouse dMDSCs by using flow cytometry. The results showed that the expression levels of both LILRBA protein and LILRBA mRNA were significantly decreased in the infected group compared with those in the uninfected group (Fig. 1a, b). The representative flow cytometry results showed that the expression of LILRB4 by the infected dMDSCs was always lower than that by the uninfected dMDSCs both in vitro and in vivo (Fig. 1c, d).

### LILRB4 expression on dMDSCs was regulated by STAT3 during *T. gondii* infection

**Fig. 2:**
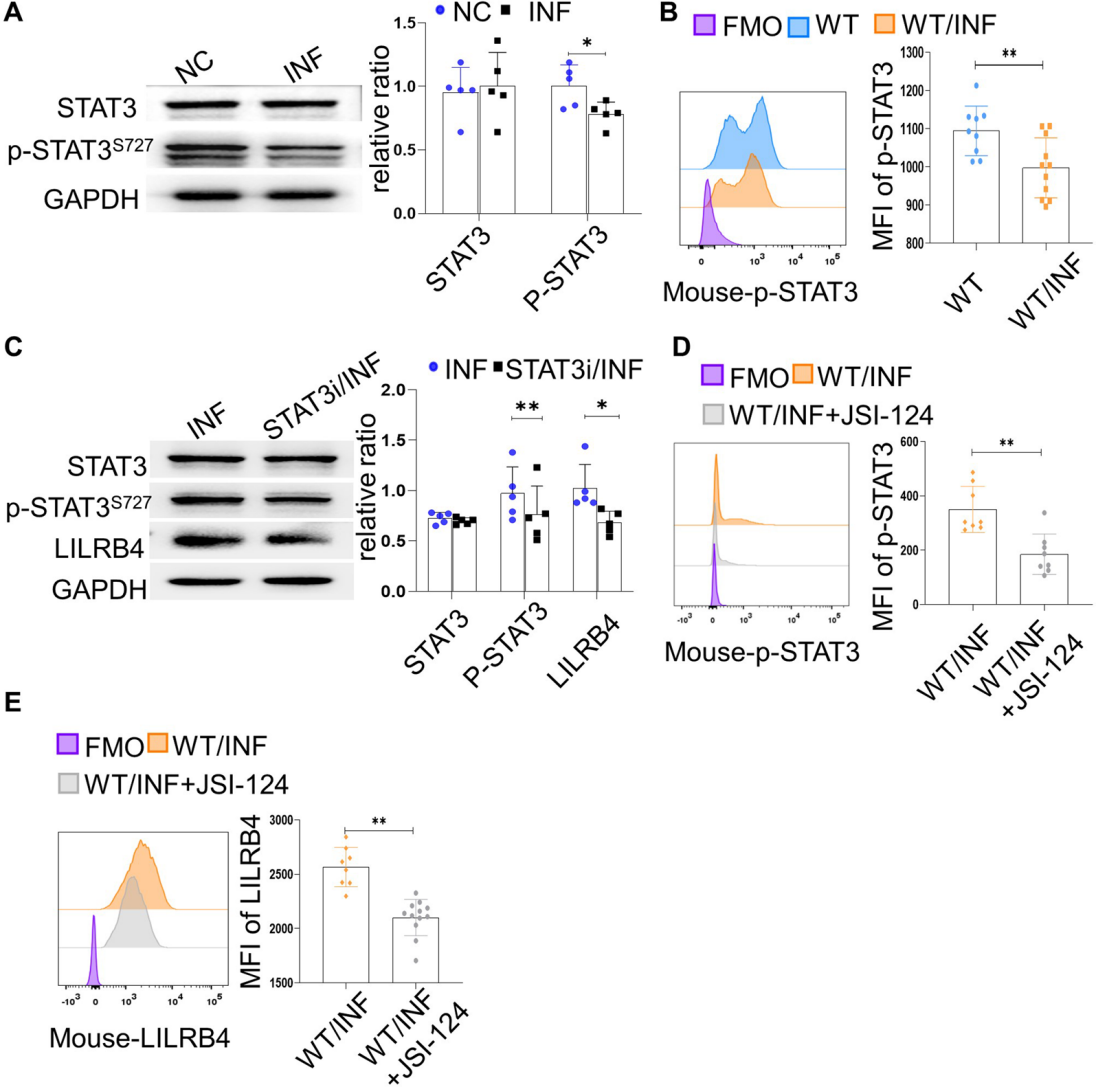
LILRB4 expression level on dMDSCs was regulated by p-STAT-3 during *T. gondii* infection. **a** Analysis of the expression of STAT3 and p-STAT3^S727^ in human dMDSCs with or without *T. gondii* infection. **b** Results of p-STAT3 expression in mouse dMDSCs of the uninfected and infected WT mice were detected by flow cytometry. **c** Expression levels of STAT3, p-STAT3^S727^ and LILRB4 in human dMDSCs treated with or without STAT3i during *T. gondii* infection were detected by Western blot. **d**, **e** Results of p-STAT3 and LILRB4 expression in mouse dMDSCs treated with or without JSI-124 during *T. gondii* infection were detected by flow cytometry. The data on human dMDSCs were identified by a paired *t*-test. The data on mouse dMDSCs were identified by the unpaired *t*-test. Asterisks indicate a significant difference at *p < 0.05 and **p < 0.01. FMO, fluorescence minus one; GAPDH, glyceraldehyde 3-phosphate dehydrogenase; INF, infected; JSI-124, the inhibitor of STAT3 *in vivo*; LILRB4, leukocyte immunoglobulin-like receptor B4; mRNA, messenger RNA; NC, negative control; p-STAT3, phosphorylated STAT3; STAT3, signal transducer and activator of transcription 3; STAT3i, the inhibitor of STAT3 *in vitro*; WT, wild type.

The expression of LILRB4 has been reported previously to be mediated by STAT3 [28]. In the present study, the p-STAT3 expression level in human dMDSCs was detected by Western blotting. The results showed that the level of p-STAT3 expression in human dMDSCs was lower in the infected group than in the uninfected group (Fig. 2a). Also, the expression level of p-STAT3 in dMDSCs of the infected mice was downregulated after *T. gondii* infection in vivo (Fig. 2b). In order to investigate whether the LILRB4 expression level on dMDSCs is regulated by STAT3, we used Static (STAT3i) and JSI-124 (STAT3i cucurbitacin I) to inhibit STAT3 phosphorylation during *T. gondii* infection both in vitro and in vivo. Human dMDSCs were analyzed by Western blotting, and mouse dMDSCs were analyzed by flow cytometry. The expressions of both p-STAT3 and LILRB4 were found to be downregulated in the infected human dMDSCs treated with STAT3i (Fig. 2c) and in the infected mouse dMDSCs treated with JSI-124 (Fig. 2d, e). These results suggested that the change in LILRB4 expression on dMDSCs after *T. gondii* infection is regulated by p-STAT3.

### The decrease in LILRB4 induced by *T. gondii* infection could further regulate the expression level of p-SHP2, p-STAT6, Arg-1 and IL-10 in dMDSCs in vitro and in vivo

**Fig. 3:**
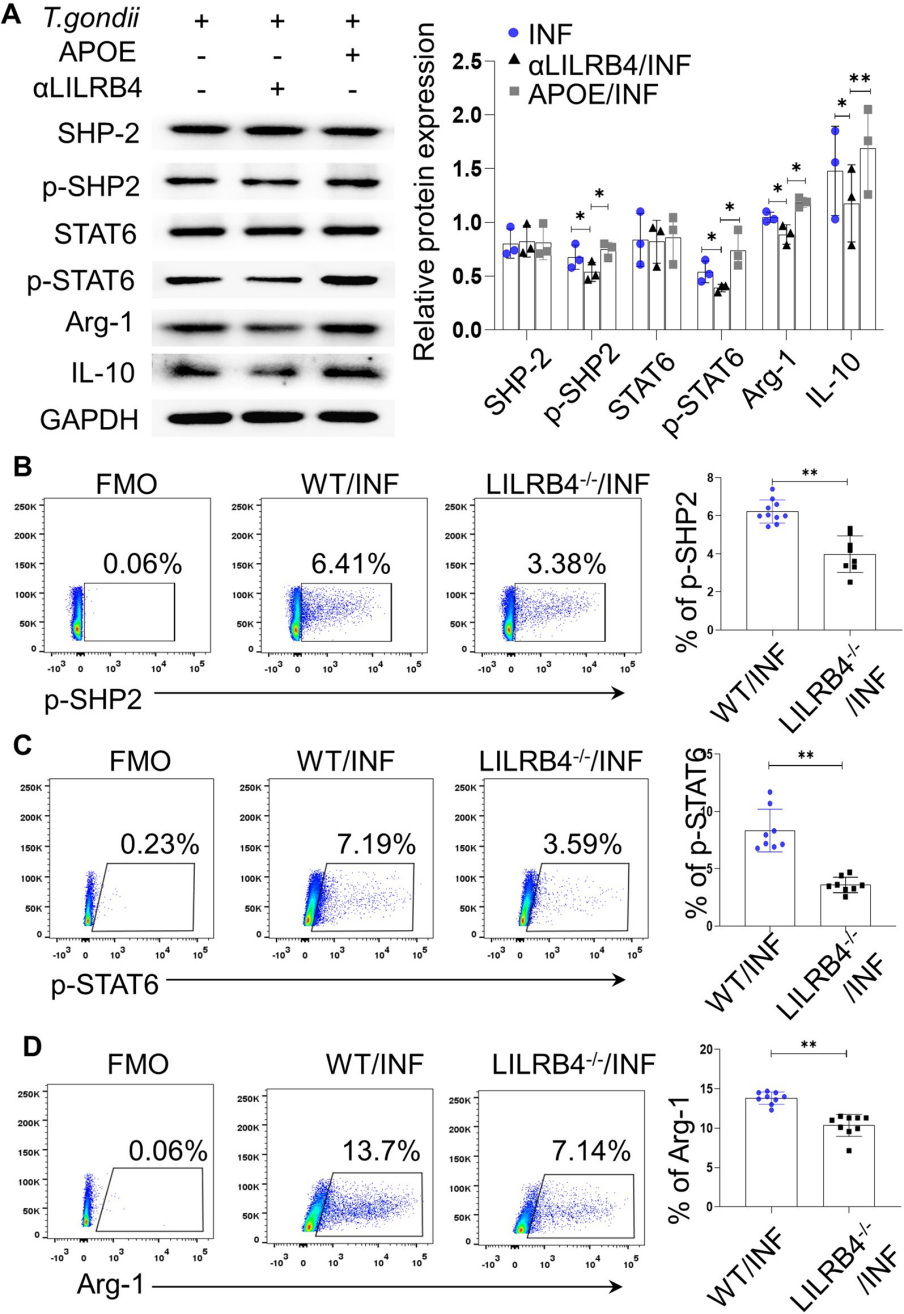
Blocking or activating LILRB4 could downregulate or upregulate the levels of p-SHP2, p-STAT6, Arg-1 and IL-10 in dMDSCs during *T. gondii* infection. **a** The expression levels of SHP-2, p-SHP2, STAT6, p-STAT6, Arg-1 and IL-10 in human dMDSCs treated with or without α-LILRB4 and APOE after infection with *T. gondii* were detected by Western blot. **b**–**d** The expression levels of p-SHP2, p-STAT6 and Arg-1 in mouse dMDSCs from the WT and LILRB4^−/−^ infected mice were detected by flow cytometry. The data on human dMDSCs were identified by a paired *t*-test. The data on mouse dMDSCs were identified by the unpaired *t*-test. Asterisks indicate a significant difference at *p < 0.05 and **p < 0.01. APOE, apolipoprotein E; Arg-1, arginase-1; FMO, fluorescence minus one; GAPDH, glyceraldehyde 3-phosphate dehydrogenase; IL-10, interleukin-10; INF, infected; LILRB4-/-, LILRB4-deficient; p-SHP2, phosphorylated SHP-2; p-STAT6, phosphorylated STAT6; SHP-2, src-homology 2 domain-containing protein tyrosine phosphatase; STAT6, signal transducer and activator of transcription 6; WT, wild type; αLILRB4: anti-LILRB4-neutralized.

As a ligand, APOE can combine directly with LILRB4 and activate it [38]. A number of authors have reported that there are two intracellular ITIMs of LILRB4 interacting with SHP-2 and activating the phosphorylation of SHP-2 [29, 30], with the SHP-2 domain recruiting STAT6 and stimulating the phosphorylation of STAT6 followed by its dimerization [31]. To explain whether the downregulation of LILRB4 on dMDSCs after *T. gondii* infection could affect the production of Arg-1 and IL-10 through the SHP-2/STAT6 pathway, we used a specific neutralizing antibody of LILRB4 or APOE to block or activate the function of LILRB4 in vitro, and the infected LILRB4^−/−^ mice with unfavorable abnormal pregnancies were established in vivo. The results showed that the phosphorylation levels of the pathway molecules (p-SHP2 and p-STAT6) and functional molecules (Arg-1 and IL-10) were decreased in the anti-LILRB4-neutralized (α-LILRB4) human dMDSCs and increased in the APOE-stimulated human dMDSCs compared with those in the infected dMDSCs (Fig. 3a). Similarly, the phosphorylation levels of pathway molecules (p-SHP2 and p-STAT6) and Arg-1 were all decreased in the infected LILRB4^−/−^ mice compared to the infected WT mice (Fig. 3B–D). Overall, our findings indicated that the change in LILRB4 induced by *T. gondii* infection might regulate the expression levels of Arg-1 and IL-10 through the SHP-2/STAT6 pathway.

### LILRB4 modulates Arg-1 and IL-10 expression via the SHP-2/STAT6 pathway in dMDSCs during *T. gondii* infection

**Fig. 4:**
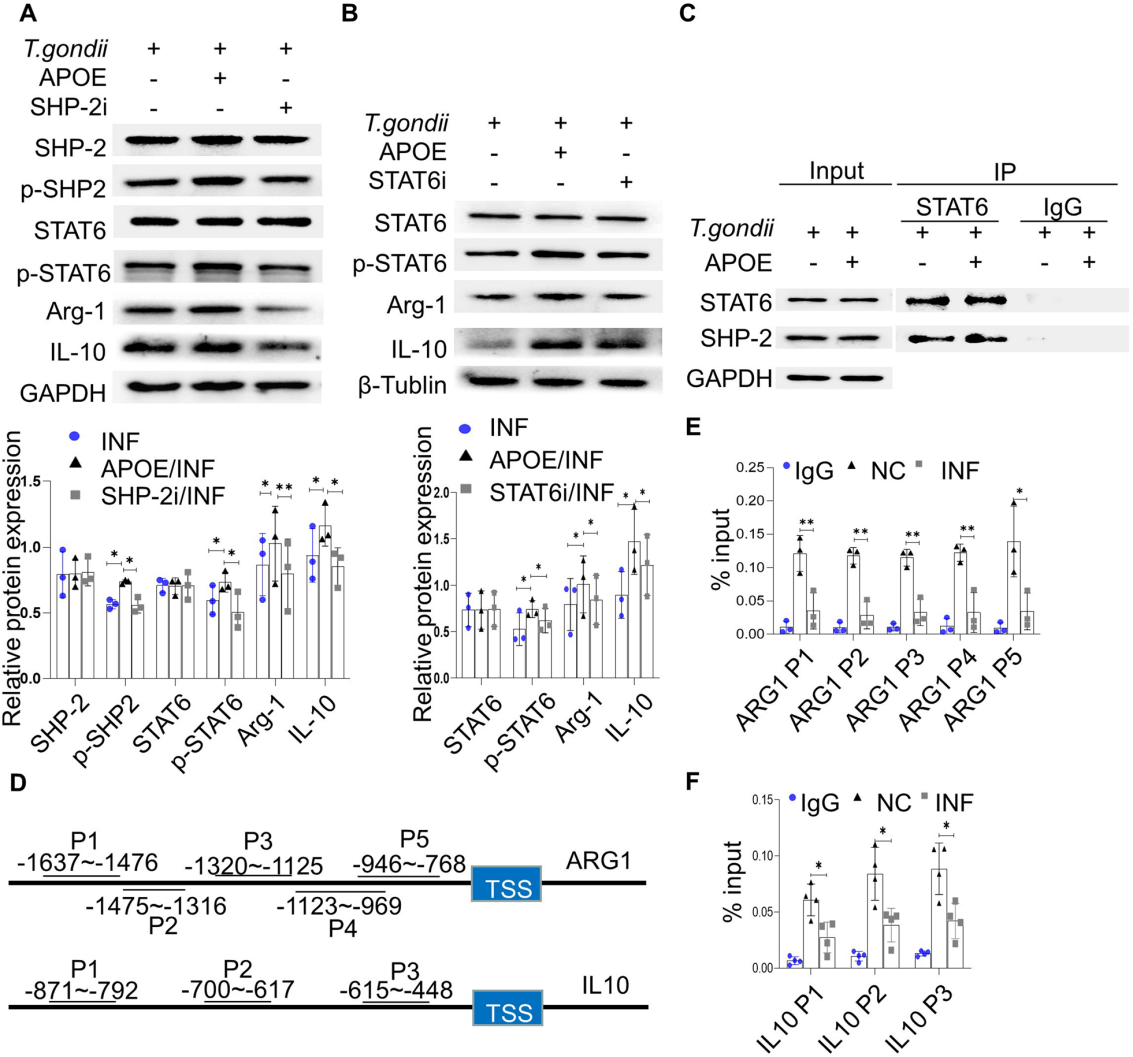
LILRB4 regulates Arg-1 and IL-10 expression levels via the SHP-2/STAT6 pathway in dMDSCs during *T. gondii* infection. **a** Expression levels of SHP-2, p-SHP2, STAT6, p-STAT6, Arg-1 and IL-10 in human dMDSCs treated with or without SHP-2i and APOE after *T. gondii* infection were detected by Western blot. **b** Expression levels of STAT6, p-STAT6, Arg-1 and IL-10 in human dMDSCs treated with or without STAT6i and APOE during *T. gondii* infection were detected by Western blot. **c** Interaction between SHP-2 and STAT6 in human dMDSCs treated with or without APOE during *T. gondii* infection was analyzed by Co-IP. **d** The schematic diagram shows the possible binding sites of STAT6 on the *ARG1* and *IL10* promoter regions. **e**–**f** ChIP-PCR of STAT6 binding regions on the promoter of *ARG1* (**e**) and *IL10* (**f**). ChIP assays were performed using anti-STAT6 and then qRT–PCR. The data of human dMDSCs were identified by a paired *t*-test. Asterisks indicate a significant difference at *p < 0.05 and **p < 0.01. APOE, apolipoprotein E; Arg-1, arginase-1;* ARG1*, the gene for Arg-1; GAPDH, glyceraldehyde 3-phosphate dehydrogenase; IgG, immunoglobulin G; IL-10, interleukin-10;* IL10*, the gene for IL-10; INF, infected; IP, immunoprecipitation; NC, negative control; P1-P5, primer 1 to primer 5; p-SHP2, phosphorylated SHP-2; p-STAT6, phosphorylated STAT6; SHP-2, src-homology 2 domain-containing protein tyrosine phosphatase; SHP-2i, the inhibitor of SHP-2; STAT6, signal transducer and activator of transcription 6; STAT6i, the inhibitor of STAT6.

To investigate whether SHP-2 can stimulate STAT6 activation, SHP099 (a selective SHP-2 inhibitor [SHP-2i]) was utilized in human dMDSCs after treatment with or without APOE during *T. gondii* infection. The results showed that p-SHP2 expression was inhibited by SHP-2i and that the phosphorylation of STAT6 was also reduced in human dMDSCs, while reduced expression levels of Arg-1 and IL-10 were observed after *T. gondii* infection (Fig. 4a). To further explore the role of STAT6 activation in regulating the expression levels of Arg-1 and IL-10 after *T. gondii* infection, human dMDSCs were treated with the STAT6 inhibitor AS1517499 (STAT6i) or APOE. The results suggested that STAT6i inhibited the activation of STAT6, whereas the production of Arg-1 and IL-10 was decreased in human dMDSCs during *T. gondii* infection (Fig. 4b). These results demonstrated that the expression levels of Arg-1 and IL-10 in dMDSCs resulting from the downregulation of LILRB4 induced by *T. gondii* infection were regulated through the SHP-2/STAT6 pathway. The Co-IP assay was carried out to further verify the interaction between SHP-2 and STAT6 in human dMDSCs treated with or without APOE after *T. gondii* infection. The SHP-2 antibody was used as a bait protein. The results showed that SHP-2 directly interacted with STAT6 in human dMDSCs during *T. gondii* infection (Fig. 4c).

STAT6 has been reported to be a transcription factor regulating the expression levels of Arg-1 and IL-10 [33, 39]. Therefore, the ChIP assay was also used to explore the activity of STAT6 binding to the promoter of *ARG1* and *IL10* in dMDSCs during *T. gondii* infection. The results showed that the enrichment of STAT6 was decreased after *T. gondii* infection in the promoter regions of *ARG1*, which span from − 1637 to− 1476 bp, from− 1475 to − 1316 bp, from − 1320 to − 1125 bp, from − 1123 to − 969 bp and from − 946 to− 768 bp (Fig. 4d, e). Moreover, the enrichment of STAT6 was reduced after *T. gondii* infection in the promoter regions of *IL10*, which span from − 871 to− 792 bp, from− 700 to − 617 bp and from − 615 to − 448 bp (Fig. 4d, f). These results suggest that STAT6 could regulate the expression levels of Arg-1 and IL-10 via binding to their respective promoters.

## Discussion

The immune microenvironment at the maternal–fetal interface plays an important role in normal pregnancy [40]. The most critical immune cells within the decidual tissue are dNK cells, Mφ, T cells, DCs and MDSCs [10, 41], and these immune cells are important for a successful pregnancy [42]. Infection by bacteria, viruses, and parasites may impair the maternal–fetal tolerance function, which can lead to fetal death, preterm birth or secondary sequelae [43, 44]. *Toxoplasma gondii* is one of the TORCH pathogens, and its infection could cause the occurrence of abnormal outcomes during pregnancy, such as spontaneous abortion, congenital retinal disease and fetal intrauterine growth retardation [45]. Our previous studies have shown that *T. gondii* infection resulted in the dysfunction of several decidual immune cells, such as Mφ [27], NK cells [8] and DCs [46]; however, it remains unknown whether *T. gondii* infection can result in the dysfunction of dMDSCs. dMDSCs have emerged as one of the novel immuno-modulators for the maintenance of maternal–fetal immune tolerance [47]. One study showed that LILRB4 expressed on MDSCs played a distinct role in inducing immunosuppression in cancer [22]. The authors of a recent study reported that LILRB4 plays a major role in regulating the immunosuppressive function of MDSCs by inhibiting the miR-1 family of microRNAs and facilitating tumor migration and invasion in the tumor microenvironment [26]. However, whether *T. gondii* infection can affect the expression of LILRB4 in dMDSCs has not been reported yet.

**Fig. 5:**
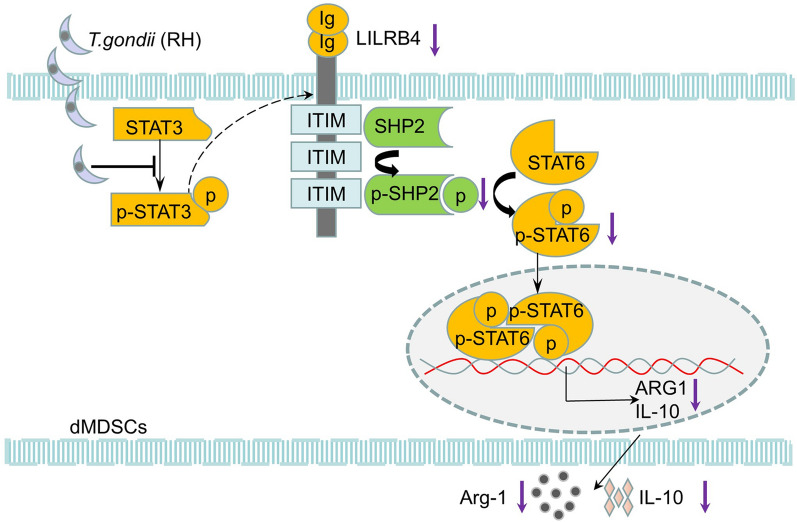
Diagrammatic representation of LILRB4 downregulation induced by *T. gondii* infection resulting in the dysfunction of dMDSCs. The phosphorylation level of STAT3 was decreased after *T. gondii* infection, resulting in the downregulation of LILRB4 on dMDSCs. The decreased level of LILRB4 reduced the phosphorylation of SHP-2 and then inhibited the activity of STAT6, which in turn inhibited the expression levels of Arg-1 and IL-10 via binding to their respective promoter during *T. gondii* infection. Arg-1, arginase-1;* ARG1*, the gene for Arg-1; dMDSCs, decidual myeloid-derived suppressor cells; Ig, immunoglobulin; IL-10, interleukin-10;* IL10*, the gene for IL-10; ITIM, immunoreceptor tyrosine-based inhibitory motifs; LILRB4, leukocyte immunoglobulin-like receptor B4; p-SHP2, phosphorylated SHP-2; p-STAT3, phosphorylated STAT3; p-STAT6, phosphorylated STAT6; SHP-2, src-homology 2 domain-containing protein tyrosine phosphatase; STAT3, signal transducer and activator of transcription 3; STAT6, signal transducer and activator of transcription 6.

To explore the change in the expression of LILRB4 on dMDSCs after *T. gondii* infection, human dMDSCs infected with *T. gondii *in vitro and infected mice with abnormal pregnancy were established in vivo. We found that the expression level of LILRB4 was clearly reduced on human dMDSCs examined by Western blotting and qPCR after infection. Similarly, the downregulation of LILRB4 was observed in infected human and mouse dMDSCs examined by flow cytometry. Thus, *T. gondii* infection can result in a decrease in the expression of LILRB4 on dMDSCs. However, the molecular mechanism of the downregulation of LILRB4 induced by *T. gondii* infection needs further exploration. In one study, the expression of LILRB4 was mediated by STAT3 phosphorylation [28], and the results of the present investigation showed that STAT3 phosphorylation in dMDSCs was reduced after *T. gondii* infection. To explore whether the downregulation of LILRB4 on dMDSCs after *T. gondii* infection resulted from weak STAT3 phosphorylation, STAT3i was used to inhibit STAT3 phosphorylation in infected human dMDSCs and infected pregnant mice. As anticipated, the expression levels of p-STAT3 and LILRB4 were all reduced when dMDSCs were treated with STAT3i during *T. gondii* infection. Therefore, the results of the present study demonstrated that the downregulation of LILRB4 in dMDSCs induced by *T. gondii* infection resulted from the inhibition of STAT3 phosphorylation (Fig. 5).

The immunosuppressive function was the essential feature of MDSCs and was due to the production of Arg-1 and IL-10 [48]. Some studies have found that the functional molecules of Arg-1 and IL-10 in MDSCs play an important role in suppressing the functions of NK and T cells, further contributing to the maternal–fetal tolerance [49, 50]. In our study, the expression of Arg-1 was downregulated in dMDSCs of LILRB4^−/−^ mice with *T. gondii* infection. Also, both Arg-1 and IL-10 in human dMDSCs were decreased or increased after the function of LILRB4 was blocked by neutralized antibody or activated by APOE under the condition of *T. gondii* infection, respectively. These results indicate that the reduction of Arg-1 and IL-10 levels in dMDSCs might be related to the downregulation of LILRB4 after *T. gondii* infection. However, while the level of LILRB4 decreased, it remains to be determined how the production of Arg-1 and IL-10 in MDSCs during *T. gondii* infection is regulated.

Some studies have suggested that LILRB4 activates the phosphorylation of SHP-2 and then stimulates the phosphorylation of STAT6 [29, 30]. In the present study, the phosphorylation of SHP-2 and STAT6 in human dMDSCs was inhibited or activated after treatment with LILRB4-neutralizing antibody or APOE, respectively, during *T. gondii* infection. In order to explore whether the reduced LILRB4 expression could affect the activation of SHP-2 and further inhibit STAT6 phosphorylation, SHP-2i was utilized to inhibit SHP-2 phosphorylation in human dMDSCs after *T. gondii* infection. This experiment revealed that SHP-2i could inhibit the phosphorylation of STAT6 after *T. gondii* infection, suggesting that STAT6 may be a downstream molecule of SHP-2. In addition, A Co-IP experiment revealed that the antibody of SHP-2 can interact with STAT6 during *T. gondii* infection. Moreover, the expression levels of Arg-1 and IL-10 were decreased after treatment with STAT6i following *T. gondii* infection. Studies found that STAT6 could regulate the expression levels of Arg-1 and IL-10 in macrophages [33, 39]. In order to observe whether STAT6 could combine with the promoters of *ARG1* and *IL10,* we performed ChIP-qPCR analyses. Our results showed that STAT6 can bind to the promoters of *ARG1* and *IL10* in dMDSCs during *T. gondii* infection.

## Conclusions

In summary, our study demonstrated that the downregulation of LILRB4 after *T. gondii* infection can regulate the expression levels of Arg-1 and IL-10 in dMDSCs through the SHP-2/STAT6 pathway. Ultimately, we revealed a novel mechanism of dMDSC dysfunction after *T. gondii* infection. This mechanism resulted in a decrease in the expression levels of Arg-1 and IL-10 via the SHP-2/STAT6 pathway, resulting in the reduction of LILRB4 levels, which may contribute to the abnormal outcomes during pregnancy.

## Supplementary Information

**Additional file 1: Fig S1.** Representative FACS gating scheme of dMDSC analyses.** A** gating strategy of human dMDSCs. After lymphocyte cells were gated by FSC-A and SSC-A, CD33+ HLA-DR- cells were gated as dMDSCs for further analyses. The FMO was used to analyze the expression of functional molecules in human dMDSCs.** B** Gating strategy of mouse dMDSCs. After lymphocyte cells were gated by FCS-A and SSC-A, CD11b+ Gr-1+ cells were gated as dMDSCs for further analysis. The FMO was used to analyze the expression of functional molecules in mouse dMDSCs.

**Additional file 2: Table S1.** Reagents and antibodies used in this study.

## Data Availability

All data generated in this study are presented within the published article.

## Abbreviations

APOE: Apolipoprotein E
Arg-1: Arginase-1
ChIP: Chromatin immunoprecipitation
Co-IP: Co-immunoprecipitation
dDCs: Decidual dendritic cells
dMφ: Decidual macrophages
dMDSCs: Decidual myeloid-derived suppressor cells
dNK: Decidual natural killer cells
FBS: Foetal bovine serum
Gd: Gestational day
HLA-DR: Human leukocyte antigen DR
IL-10: Interleukin-10
LILRB4: Leukocyte immunoglobulin-like receptor B4
p-SHP2: Phosphorylated SHP-2
p-STAT3: Phosphorylated STAT3
p-STAT6: Phosphorylated STAT6
PBS: Phosphate buffered saline
qRT-PCR: Quantitative reverse transcription PCR
SHP-2: Src-homology 2 domain-containing protein tyrosine phosphatase
STAT3: Signal transducer and activator of transcription 3
STAT6: Signal transducer and activator of transcription 6
