# Supplementary figures and images for: LILRB4 regulates the function of decidual MDSCs via the SHP-2/STAT6 pathway during Toxoplasma gondii infection
Source: Parasit Vectors. 2023 Jul 17;16:237. doi: 10.1186/s13071-023-05856-4 (PMC10353217; doi:10.1186/s13071-023-05856-4)

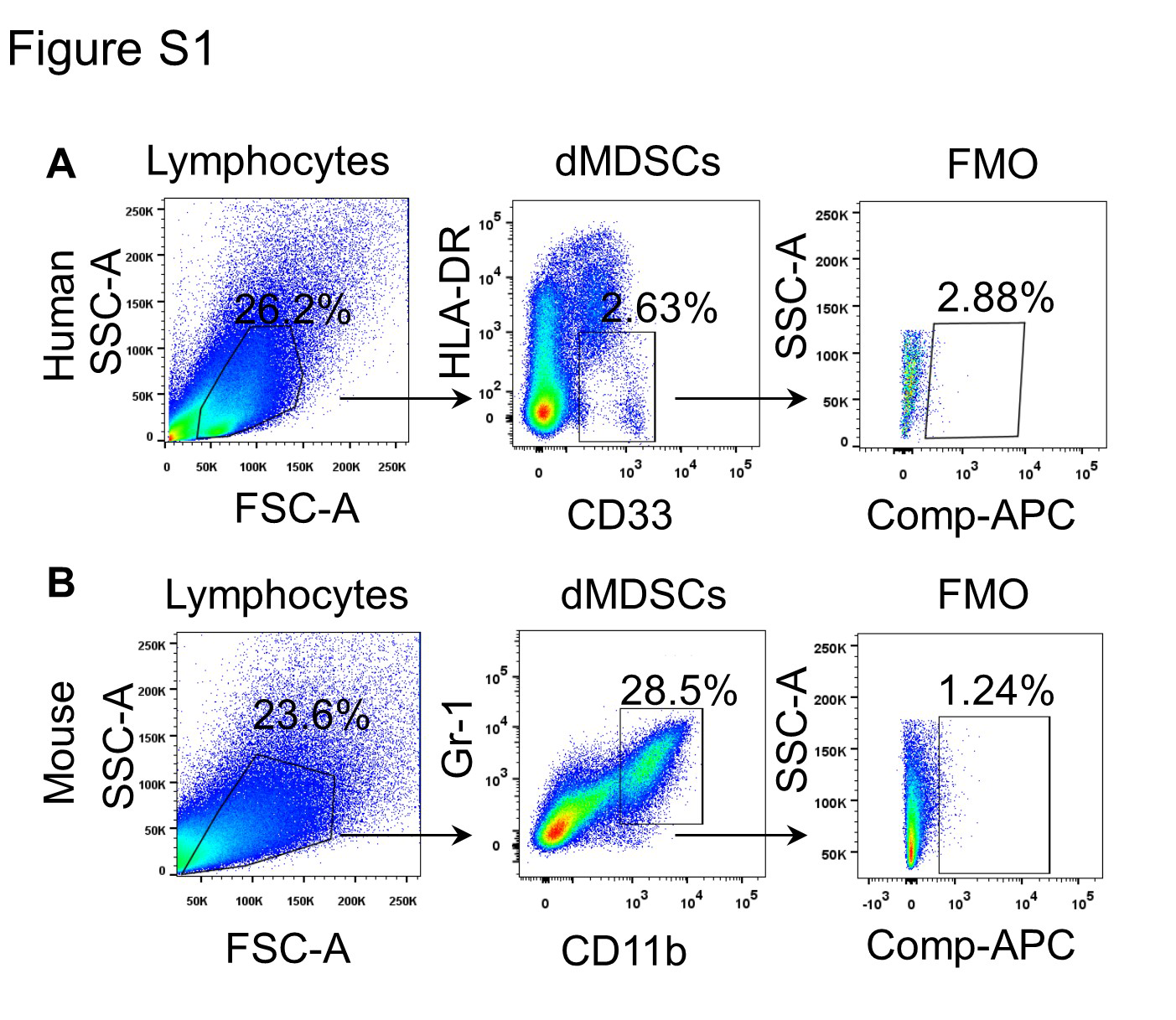

Supplement: Supplementary file 1 — Additional file 1: Fig S1. Representative FACS gating scheme of dMDSC analyses. A gating strategy of human dMDSCs. After lymphocyte cells were gated by FSC-A and SSC-A, CD33+ HLA-DR- cells were gated as dMDSCs for further analyses. The FMO was used to analyze the expression of functional molecules in human dMDSCs. B Gating strategy of mouse dMDSCs. After lymphocyte cells were gated by FCS-A and SSC-A, CD11b+ Gr-1+ cells were gated as dMDSCs for further analysis. The FMO was used to analyze the expression of functional molecules in mouse dMDSCs. [file 13071_2023_5856_MOESM1_ESM.tif]
