# Supplementary material for: LILRB4 regulates the function of decidual MDSCs via the SHP-2/STAT6 pathway during Toxoplasma gondii infection
Source: Parasit Vectors. 2023 Jul 17;16:237. doi: 10.1186/s13071-023-05856-4 (PMC10353217; doi:10.1186/s13071-023-05856-4)
Supplement: Supplementary file 2 — Additional file 2: Table S1. Reagents and antibodies used in this study. [file 13071_2023_5856_MOESM2_ESM.docx]

Table S1 Reagents and antibodies used in this study

| **REAGENT or RESOURCE** | **SOURCE** | **IDENTIFIER** |
| --- | --- | --- |
| **Antibodies** | | |
| Anti-GAPDH (CloneNo.1E6D9) human | Protientech | Cat No.:60004-1-Ig |
| Anti-Arg-1 (CloneNo.5D6D12) human | Protientech | Cat No.:66129-1-Ig |
| Anti-ILT-3 antibody human | Abcam | Cat No.:ab229749 |
| Anti-IL-10 human | Wanlei | Cat No.: WL03088 |
| Anti-STAT6 (CloneNo.YE361) human | Abcam | Cat No.:ab32520 |
| Stat6 Rabbit mAb (CloneNo.D3H4) human | Cell Signaling Technology | Cat No.:5397S |
| Anti-STAT6 (phosphoY641) (EPR18278-265) human | Abcam | Cat No.:ab188080 |
| Ant-STAT3 (CloneNo.3G2D12) human | Protientech | Cat No.:60199-1-Ig |
| Ant-P-STAT3 (phosphoS727) (CloneNo.E121-31) human | Abcam | Cat No.:ab32143 |
| Anti-SHP-2 human | Protientech | Cat No.:20145-1-AP |
| Anti-Phospho-SHP2 (Tyr580) human | Bioss | Cat No.: bs-3405R |
| Anti-Rabbit IgG HRP | Abmart | Cat No.: M21006 |
| SimpleChIP® Enzymatic Chromatin IP Kit (Agarose Beads) | Cell Signaling Technology | Cat No.: 9002S |
| ProteinA/G Immunoprecipitation Kit | Solarbio | Cat No.: M2410 |
| Anti-β-tubulin | Protientech | Cat No.: 10094-1-AP |
| Rabbit Control IgG | ABclonal | Cat No.:AC005 |
| LILRB4 Monoclonal Antibody (ZM4.1) | Invitrogen | Cat No.:16-5139-38 |
| SHP099 (SHP-2 inhibitor ) | MCE | Cat No.:HY-100388 |
| AS1517499（STAT6 inhibitor） | MCE | Cat No.:HY-100614 |
| Stattic (STAT3 inhibitor) human | MCE | Cat No.:HY-13818 |
| Cucurbitacin Ⅰ (STAT3 inhibitor) mouse | MCE | Cat No.:HY-N1405 |
| Recombinant Human ApoE | Novoprotein | Cat No.:DC102 |
| PerCP/Cy5.5-CD11b (M1/70) human/Mouse | Biolegend | Cat No.:101227 |
| FITC-Gr-1 (RB6-8C5) mouse | Biolegend | Cat No.:108405 |
| APC-Arg-1 (A1exF5) human/mouse | Invitrogen | Cat No.:17-3697-82 |
| PE-anti-CD85k (gp49 Receptor) Antibody mouse | Biolegend | Cat No.:144904 |
| BV421-P-STAT3 (pS727) (49/p-Stat3) human/Mouse | BD | Cat No.:565416 |
| APC-p-SHP2 (Tyr580) (Shp2Y580-4A2) human/Mouse | Invitrogen | Cat No.:MA5-37264 |
| APC-p-STAT6 (Tyr641) (CHI2S4N) human/Mouse | Invitrogen | Cat No.:17-9013-41 |
| PE-HLA-DR (L243) human | Biolegend | Cat No.:307605 |
| PE-CD33 (WM53) human | Biolegend | Cat No.:303404 |
| PerCP/Cy5.5-HLA-DR (L243) human | Biolegend | Cat No.:307630 |
| PerCP/Cy5.5-CD33 (P67.6) human | Biolegend | Cat No.:366616 |
| APC-LILRB4(ZM4.1)human | Invitrogen | Cat No.:17-5139-42 |
| **Primers for qPCR** | | |
| Human GAPDH-Fs | Sangon Biotech | 5’-TCAACGGCACAGTCAAGG-3’ |
| Human GAPDH-Rs | Sangon Biotech | 5’-TACTCAGCACCGGCCTCA-3’ |
| Human LILRB4-Fs | Sangon Biotech | 5’-ACCAGGCTGAATTCCCCATG-3’ |
| Human LILRB4-Rs | Sangon Biotech | 5’-GAGGAGGGAGAGAAGCAGGA-3’ |
| **Primers for ChIP-qPCR** | | |
| Arg-1 Primer1-Fs | Sangon Biotech | 5’-TCAATGGGTGTTGTTGAGGA-3’ |
| Arg-1 Primer1-Rs | Sangon Biotech | 5’-CCCCATCAAATTGTAGGTATGT-3’ |
| Arg-1 Primer2-Fs | Sangon Biotech | 5’-TGGCAGGAATTTAATAAGACTTCC-3’ |
| Arg-1 Primer2-Rs | Sangon Biotech | 5’-TAACCTTATAGCATTGACATTGCAA-3’ |
| Arg-1 Primer3-Fs | Sangon Biotech | 5’-GGTTAATATCTAGGCAATATGAGGAA-3’ |
| Arg-1 Primer3-Rs | Sangon Biotech | 5’-GGGCCTAGAAGAGTGTTGGTG-3’ |
| Arg-1 Primer4-Fs | Sangon Biotech | 5’-GGGGATACAGCAGACAAAATTC-3’ |
| Arg-1 Primer4-Rs | Sangon Biotech | 5’-GCACCTTAAAGATCCTTCCGA-3’ |
| Arg-1 Primer5-Fs | Sangon Biotech | 5’-TGTATGGTGACAAATGGTAGCTC-3’ |
| Arg-1 Primer5-Rs | Sangon Biotech | 5’-TTGGTGCCATTATACTGATGAATAA-3’ |
| IL-10 Primer1-Fs | Sangon Biotech | 5’-CTGGAGATGGTGTACAGTAGGG-3’ |
| IL-10 Primer1-Rs | Sangon Biotech | 5’-GCACTTGCTGAAAGCTTCTTATA-3’ |
| IL-10 Primer2-Fs | Sangon Biotech | 5’-CTCTGTGCCTCAGTTTGCTC-3’ |
| IL-10 Primer2-Rs | Sangon Biotech | 5’-TTAGGATTCTCAGGCACATGTT-3’ |
| IL-10 Primer3-Fs | Sangon Biotech | 5’-GAAATCGGGGTAAAGGAGC-3’ |
| IL-10 Primer3-Rs | Sangon Biotech | 5’-CAGTCACCTTAGGTCTCTGGG-3’ |
